# Genetic tools for engineering *Zymomonas mobilis*,* Cereibacter sphaeroides* and *Novosphingobium aromaticivorans* to improve production of bioenergy compounds

**DOI:** 10.1186/s12934-025-02845-3

**Published:** 2025-11-14

**Authors:** Shikha Mishra, Vishwajit Kumar, Jyotsna Misra, Amritha K.P., Balendra Sah, Piyush Behari Lal

**Affiliations:** 1 Center for Emerging and Tropical Diseases, Kasturba Medical College, Manipal, Manipal, India; 2 Manipal Academy of Higher Education, Manipal, Karnataka 576104 India; 3 Department of Microbiology, Kasturba Medical College, Manipal, Academy of Higher Education, Manipal, 576104 Karnataka India; 4 Department of Biotechnology, Graphic Era Deemed to be University, Dehradun, 248002 Uttarakhand India; 5 Manipal School of Life Sciences, Manipal Academy of Higher Education, Manipal, 576104 Karnataka India; 6 DOE, Great Lakes Bioenergy Research Center, University of Wisconsin-Madison, WI, 53706 USA

**Keywords:** Biofuel, Metabolic engineering, *Zymomonas mobilis*, *Novosphingobium aromaticivorans*, *Cereibacter sphaeroides*

## Abstract

Limited genetic tools for non-model bacteria are one of the limiting factors for genetic studies. This review compiles genetic tools used for three non-model alpha-proteobacteria, such as *Zymomonas mobilis*, *Cereibacter* (*Rhodobacter) sphaeroides*, and *Novosphingobium aromaticivorans*, which hold significant potential to produce industrially essential bioenergy compounds due to their distinctive metabolic pathways and resilience in extreme environments. Each of these strains has a unique genetic profile that enables them to efficiently carry out key reactions relevant to producing bioenergy compounds, such as converting sugars into bioenergy compounds and breaking down lignotoxins. Genetic tools can further optimize these strains for enhanced bioenergy compound production. This review explores the metabolic advantages of these organisms. It highlights the available array of genetic toolkits that can be shared among them to unlock their full potential for sustainable biofuel production.

**Graphical abstract:**

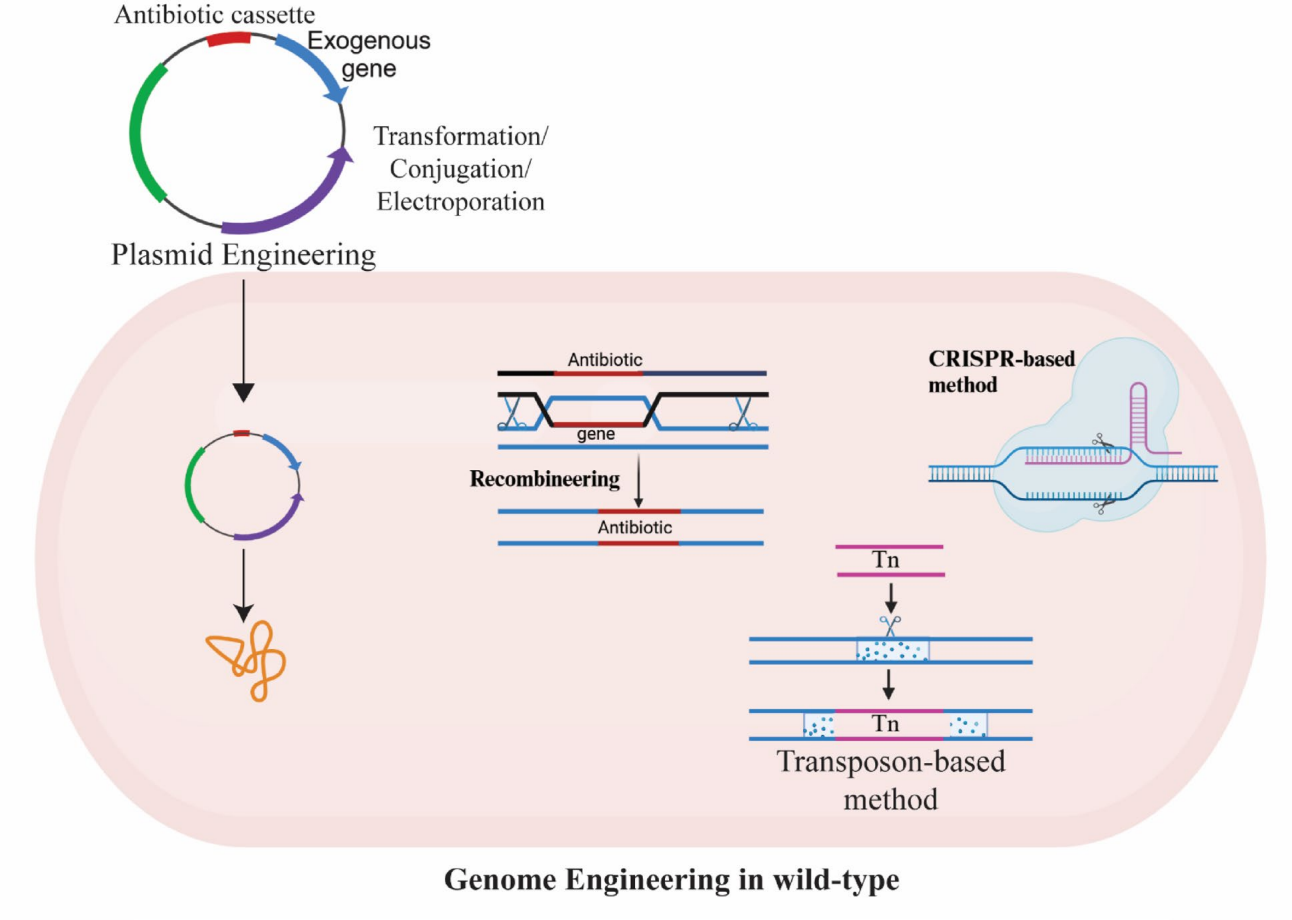

**Supplementary Information:**

The online version contains supplementary material available at 10.1186/s12934-025-02845-3.

## Introduction

This review highlights the genetic tools and engineering strategies developed for three metabolically versatile Alphaproteobacteria, *Zymomonas mobilis*, *Cereibacter* spp., and *Novosphingobium spp*., with a focus on their potential in the production of bioenergy compounds. *Z. mobilis* is a model ethanologen known for its high ethanol yield [1]. *Cereibacter sphaeroides* (formerly *Rhodobacter sphaeroides*) contributes to biomass breakdown, CO_2_ fixation, and terpene production [2, 3], and *N. aromaticivorans* is notable for degrading lignin and aromatic compounds [4]. Each microbe has unique genetic capabilities that, when combined, provide a strong foundation for producing a diverse range of sustainable bio-based compounds. Although these species possess strong native metabolisms, they require genetic enhancements to expand their substrate range, increase product yields, and improve industrial stress tolerance, necessitating advanced genetic tools. Their close phylogenetic relationship suggests that genetic tools developed for one species could be adapted for others, accelerating strain engineering. Accordingly, this review evaluates the current genetic toolkits available for each organism, emphasizing opportunities for cross-species tool sharing.

### *Zymomonas mobilis*

*Z. mobilis* exhibits several characteristics that make it an excellent host for ethanol production. Characteristics of *Z. mobilis* have been thoroughly studied and documented in several reviews [5, 6]. A few highlights are its high specific glucose uptake rate [7], elevated ethanol titer, and robust ethanol tolerance that enables its metabolic engineering for bioethanol synthesis from renewable substrates, particularly lignocellulose hydrolysates [8, 9]. The strain utilizes the Entner–Doudoroff (ED) pathway, which employs fewer enzymatic steps and produces one ATP, one NADH, and two molecules of pyruvate per mole of glucose (Fig. S1) [10]. The lower ATP yield makes *Z. mobilis* less energy-efficient, which is linked to its rapid glucose uptake and ethanol production rates. It helps the strain to convert 98% of substrate carbon into ethanol [9]​, generating minimal biomass waste in the bioreactor.

Significant research has been conducted on *Z. mobilis*, focusing on developing genetic tools, metabolic engineering strategies, bioproduct production, and genomic and transcriptomic analysis [1, 11, 12]. This bacterium’s small 2.1 Mb genome, organized into a circular chromosome and four plasmids, facilitates genetic modification over other species with large genomes [13]. The metabolic analysis via phenotypic microarray provides a detailed datasets for the utilization of glucose, fructose, and various nitrogen, phosphorus, and sulfur sources, tolerating acidic pH (down to 4.0) and resisting many inhibitors, and showing sensitivity to chloride and nitrite [14]. The genome annotations [13] and phenotypic microarray datasets [14] have further enhanced the genetic understanding of *Z. mobilis*, enabling systematic studies for the biofuel synthesis pathway. Consequently, several metabolic models have been published to date, including a medium-level stoichiometric model for flux balance [15], a kinetic model simulating glycolysis *in vivo* and cell-free extract [16], and six genome-scale metabolic models [7, 17–19]. These models highlighted the importance of the ED pathway, pyruvate decarboxylase, and two alcohol dehydrogenase isoenzymes in the fermentative production of ethanol and carbon dioxide from glucose. Metabolic control analysis of the model indicated that most flux control resides predominantly in ATP consumption [16]. The recent two models of *Z. mobilis*, iZM4_478 [20] and iHN446 [7], involve various genes, reactions, and metabolites. The iHN446 model was refined using gene annotations, literature, physiological data, and biochemical databases [7]. The gene essentiality predictions in iZM4_478 are based mainly on the phenotypic data of the individual pooled transposon insertion mutants [20]. Both models are based on mutant phenotypes generated through random transposon mutagenesis. Transposon mutagenesis, combined with high-throughput sequencing, generates extensive datasets that help assess mutant fitness under diverse environmental conditions, providing valuable insights for refining genetic and metabolic models of *Z. mobilis* [21]. These insights are crucial for optimizing genetic modifications, including integrating exogenous pathways to enhance bioenergy production from agricultural waste. Transposon mutagenesis, combined with high-throughput sequencing, generates extensive datasets that help assess mutant fitness under diverse environmental conditions, providing valuable insights for refining genetic and metabolic models of *Z. mobilis* [21]. These insights are crucial for optimizing genetic modifications, including integrating exogenous pathways to enhance bioenergy production from agricultural waste. Recent advancements in plasmid development have further facilitated such modifications, enabling *Z. mobilis* strains to efficiently convert diverse carbohydrates into valuable biofuels, as discussed in the tools section. While *Z. mobilis* shows remarkable capabilities, these limitations highlight the need for further research and development to maximize its capabilities in biotechnological production [22].

### *Cereibacter sphaeroides*

*Cereibacter sphaeroides* (*C. sphaeroides*) is a purple non-sulfur alpha-proteobacterium from the *Rhodobacteraceae* family [23, 24]. The genome of *C. sphaeroides* is well-characterized at 4.5 Mb, which includes two chromosomes (CI and CII) and five additional replicons [25]. This bacterium has potential for biosynthesizing industrially relevant compounds such as terpene-based bioenergy compounds like bisabolene [26], terpene-based fuels [27], bioplastics [28], etc. Terpenes are synthesized by their native MEP (2-methyl-d-erythritol 4-phosphate) pathway. Strains are also constructed with heterologous growth uncoupled MVA (mevalonate) pathway, redirecting its metabolic resources to isoprenoid synthesis [29] and making the strains an efficient chassis for isoprenoid synthesis, a precursor molecule for terpene-based bioenergy compounds (Fig. S2). One of the notable features of this bacterium is the ability to perform anoxygenic photosynthesis under anaerobic conditions using light energy [30]. Its ability to convert light energy into chemical energy, metabolic versatility, and utilize various organic compounds as electron donors (such as succinate or malate) instead of water makes it an attractive candidate for the biosynthesis of bioenergy compounds [31]. Its metabolic flexibility allows it to adapt to environmental conditions such as light availability, oxygen, and nutrients [32–34].

*C. sphaeroides* has great potential for bioenergy compounds production, but fully leveraging this requires precise metabolic engineering to optimize pathway fluxes [35]. A key example is the Calvin–Benson–Bassham (CBB) pathway, which plays a critical role in converting carbon sources into biomass and energy-rich compounds. This pathway is regulated by the transcription factor CbbR. In *C. sphaeroides*, similar to related species such as *Rhodobacter capsulatus*, metabolic signals from enzymes like phosphoribulokinase influence CbbR activity, thereby modulating the flux through the CBB cycle [36]. Fine-tuning this regulation can enhance carbon assimilation and channel metabolic pathways toward bioenergy production. Therefore, understanding and engineering CbbR-mediated gene expression is essential for boosting bioenergy yields in *C. sphaeroides*.

*C. sphaeroides* has been extensively studied for photoheterotrophy, nitrogen fixation, and carbon fixation [37, 38]. Its natural ethanol-producing yield is lower than that of other bacterial species, but co-culture studies with closely related species *R. capsulatus* and *Chlamydomonas reinhardtii* have been shown to enhance ethanol yield. While *C. sphaeroides* can switch to fermentation under anaerobic conditions, yielding terpene-based bioenergy compounds, metabolic engineering is required to harness light energy to produce bioenergy compounds by modifying enzymes and pathways to optimize redox balance and improve yields [27, 39, 40]. The metabolic mechanisms involved in phototrophy, respiration, and various other metabolic pathways have been analyzed [37], but further improvements are needed. For example, the most prominent and widely utilized model for *C. sphaeroides* is the genome-scale metabolic network reconstruction known as iRsp1095, first published in 2011. This model incorporates 1,095 genes, 796 metabolites, and 1,158 reactions, and features biomass reactions specific to *C. sphaeroides* [41] *and* iRsp1140, which maps *C. sphaeroides* metabolism using 1,140 genes, 878 metabolites, and 1,416 reactions. It provides insights into pyridine nucleotide cycling during aerobic respiration and anaerobic photosynthesis, aiding biofuel research [42].

These insights highlight *Cereibacter sp.* as a promising candidate for utilizing light energy and aiding bioenergy compound production. However, the strain requires further optimization to achieve a high yield of bioenergy compounds, such as by fine-tuning gene expression to enhance flux from key pathways, such as carbohydrate metabolism and the Calvin-Benson-Bassham pathway (CO_2_ fixation), to the MEP or MVA pathway. This process necessitates efficient genetic tools for strain improvement, which are discussed in this review.

### *Novosphingobium aromaticivorans*

**Fig. 1:**
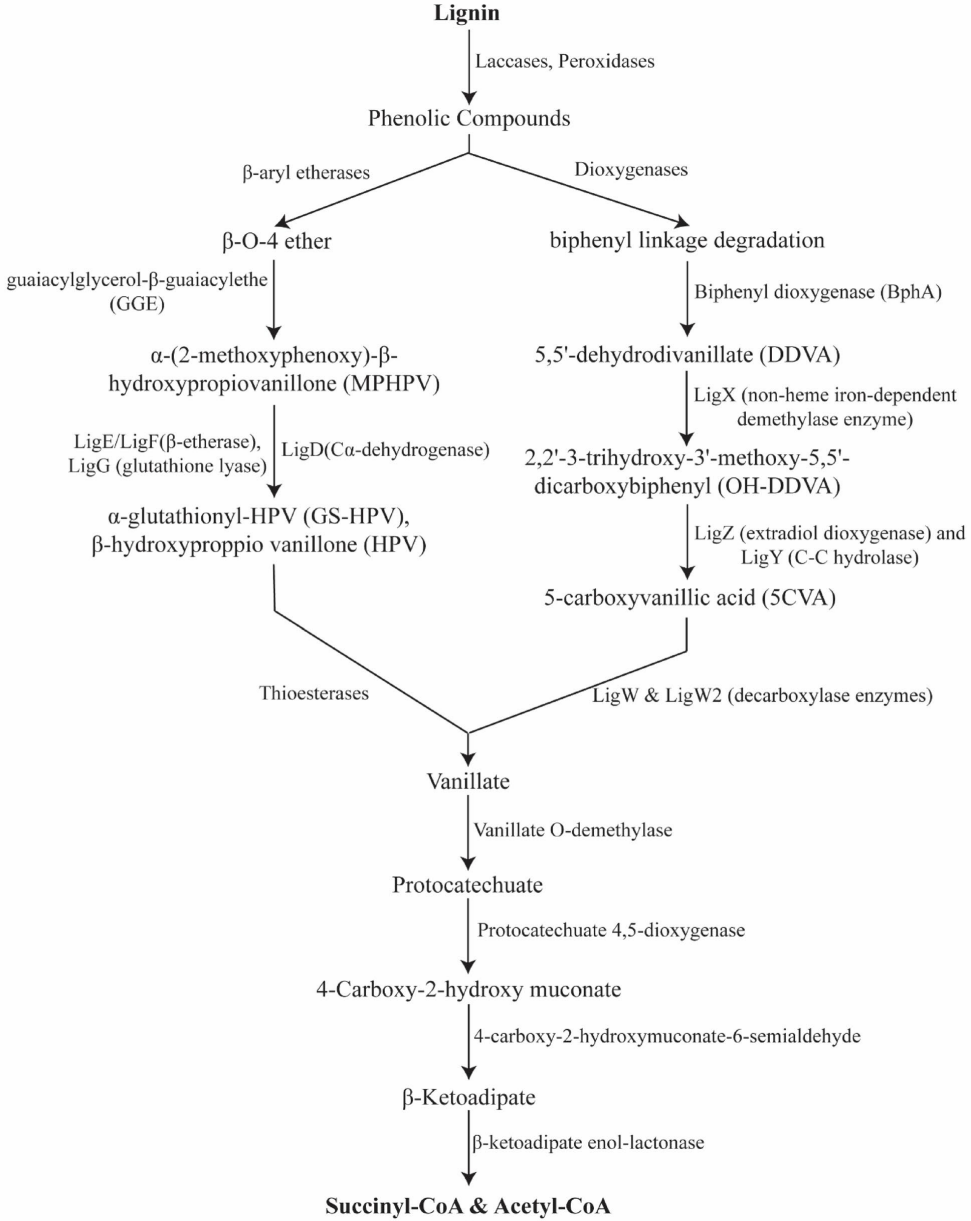
Lignin degradation pathway in *Novosphingobium* sp. The pathway highlights the bacterium’s ability to convert lignin into metabolically beneficial compounds, demonstrating its potential for aromatic compound degradation

The genus *Novosphingobium*, a rod-shaped, gram-negative alpha-proteobacterium, found in deep terrestrial and subsurface sediments, is known for its metabolic versatility [43, 44], particularly in degrading aromatic compounds, hydrocarbons, including alkanes and aromatic compounds like benzene, toluene, ethylbenzene, xylene, and polycyclic aromatic hydrocarbons [45]. Additionally, its lignin degradation pathway [4] generates key metabolic intermediates such as succinyl-CoA, acetyl-CoA, and vanillic acid, as illustrated in Fig. 1 [46]. Several strains, including *Novosphingobium piscinae* (*N. piscinae*) [47], *Novosphingobium rhizosphaerae* (*N. rhizosphaerae*) [48], *Novosphingobium taihuense* (*N. taihuense*) [49], *Novosphingobium subterraneum* (*N. subterraneum*) [50], *Novosphingobium aromaticivorans* (*N. aromaticivorans)* [50], and *Novosphingobium capsulatum (N. capsulatum)* [51], are of interest for biotechnological applications, along with recently discovered strains like UCM-28T [52] and E-II-3(T) [53]. The 27 *Novosphingobium* strains had an average genome size of 4.97 Mbp [54]. This bacterium has potential for biofuel applications, particularly in hydrocarbon biodegradation and biomass conversion.

It is a well-characterized lignin-degrading bacterium known for its ability to catabolize a broad range of aromatic compounds derived from lignin. Lignin biotransformation is facilitated by specialized enzymes that orchestrate the breakdown and assimilation of various lignin-derived aromatic compounds. These enzymes include oxidative catalysts, such as dioxygenases (e.g., *LigAB*), which mediate aromatic ring cleavage, and LigE, which specifically targets the *β-O-4* ether bond. Other enzymes perform O-demethylation and hydroxylation reactions, enabling the assimilation of diverse lignin-derived fragments into central metabolic pathways, along with several additional genes summarized in Table 1. The combined action of these enzymes allows *Novosphingobium* to efficiently metabolize and valorize a broad spectrum of lignin derivatives, making it a promising chassis for lignin bioconversion and sustainable bioproduct synthesis [55–58].

**Table 1:** Table showing the list of genes involved in lignin degradation in *N. aromaticivorans*

S. No.	Gene with Accession No.	Enzyme Name	Activity / Bond Cleaved	Description
1.	*DypA* (Saro_2861; cl46457)	DyP-type peroxidase	Oxidative cleavage of lignin bonds (β-O-4, C-C)	Broad activity; cleaves both phenolic and non-phenolic units
2.	MCO (Saro_2876; pfam01565)	Multicopper oxidase (Laccase)	Oxidation of phenolic lignin structures (β–O–4 modification, polymerization)	Acts indirectly on lignin structure; not bond-specific cleavage
3.	Xylanase (Saro_1884; cd18622)	glycoside hydrolase	hydrolyze the β-1,4-xylosidic bonds	hydrolyze the β-1,4-xylosidic bonds in xylan, a key hemicellulose in plant cell walls, converting it into xylose and xylo-oligosaccharides
4.	*LigA* (Saro_1233; cd07925)	NAD⁺-dependent dehydrogenase	Converts guaiacylglycerol-β-guaiacyl ether (GGE) into a ketone form	Prepares β–O–4 linkage for further cleavage
5.	*LigB* (Saro_1234; cd07949)	β-etherase	β–O–4 ether bond cleavage (with glutathione)	Works with LigE and LigF in a multi-step process
6.	*LigE* (Saro_2405; cd03202)	Glutathione S-transferase	β–O–4 ether bond cleavage (aromatic end)	Acts in tandem with LigF
7.	*LigF* (Saro_2404; cl43132)	Glutathione S-transferase	β–O–4 ether bond cleavage (non-phenolic end)	Enantioselective partner to LigE
8.	*LigG* (Saro_2403; PRK13449)	Glutathione lyase	Removes glutathione from ether cleavage products	Final step in β–O–4 cleavage pathway
9.	*LigZ* (Saro_2804; COG1846)	Aromatic ring-hydroxylating dioxygenase	Cleaves aromatic rings after β–O–4 bond cleavage	Involved in downstream catabolism
10.	*DesA* (Saro_2404; pfam00903)	Syringate O-demethylase	Demethylation of syringyl units	Important for S-unit processing (aromatic modification)
11.	*LigC* (Saro_2836; pfam02537)	Glutathione S-transferase	β–O–4 ether bond cleavage (similar to LigF/LigE)	Functional redundancy in β–O–4 cleavage

This strain employs a unique glutathione S-transferase (Nu-class GST), which acts as a glutathione lyase to cleave β-aryl ether bonds, the most abundant linkages in lignin, through deglutathionylation of glutathione–β-hydroxypropiovanillone conjugates (GS-HPV) [59]. Experimental evolution and mutational studies have further uncovered enzymes responsible for oxidizing lignin-derived intermediates such as guaiacylglycerol-β-guaiacyl ether (GGE), with improved expression of aldehyde dehydrogenases contributing to enhanced aromatic catabolism [60]. Genomic studies of strains like *N. aromaticivorans* MBES04 also reveal genes encoding vanillate monooxygenase, protocatechuate 4,5-dioxygenase, and catechol degradation pathways, supporting its versatile lignin valorization potential [61].

*Novosphingobium species* is predicted bioinformatically to have pathways for ethanol synthesis via the EMP pathway (KEGG pathway- nar00010) xylose utilization via the pentose phosphate pathway (KEGG pathway- nar_M00007), and terpenoid synthesis via the MEP pathway (KEGG pathway- nar00900). *N. aromaticivorans sp.* is shown for converting complex plant materials into fermentable sugars by cellulases, hemicellulases, and lignin-degrading enzymes [62, 63], and for carotenoid synthesis from aromatic compounds [64]. *Novosphingobium* may work synergistically with other microorganisms in the degradation of complex sugars and fermentation to enhance the yield of end products [65]. This cooperative behaviour enhances overall degradation efficiency and supports sustainable waste management and biomass utilization approaches.

*Novosphingobium* spp. exhibit notable metabolic flexibility, including nitrogen fixation [66] and tolerance to pH fluctuations [67], temperature shifts [68], and toxic compounds [62, 69], making them attractive for biotechnological applications such as oil spill remediation [70]. These bacteria employ enzymes like hydroxylases and dioxygenases to degrade complex hydrocarbons into more biodegradable forms [71]. Their metabolic potential has been explored through models like iNOVO479, which integrates 479 genes, 645 reactions, and 604 metabolites, offering a foundational framework for studying *N. aromaticivorans* [72]. However, further experimental data are needed to improve model predictions for growth, biomass yield, and product formation under diverse conditions.

Bioinformatic analyses have unveiled the presence of putative lignin-degrading genes in *Novosphingobium spp.*, highlighting their latent potential in valorizing lignin-derived aromatic compounds (Manuscript in preparation). However, the field now demands a paradigm shift from in silico predictions to functional exploration and synthetic repurposing of these genetic elements. Future research should strategically pivot toward harnessing and engineering these pathways in tractable chassis or through genome-enabled strain improvement, thereby unlocking novel avenues for lignin valorization in biorefinery applications. Despite its promise, this bacterium remains unexplored mainly for renewable fuel synthesis. The strain needs to be tailored genetically for efficient use in the biosynthesis of bioenergy compounds. The facile method and tools discussed in this review will be helpful in genetics in *Novosphingobium sp.*

### Recent advances in genetic engineering tools for *Zymomonas mobilis*, *Cereibacter Sp.*, and *Novosphingobium Sp.*

Genetic engineering tools are specialized molecular techniques used to directly modify organisms’ genetic material (DNA or RNA), enabling the precise addition, removal, or alteration of genes. These tools are fundamental for studying gene function, enhancing traits, and developing genetically modified organisms (GMOs) for diverse applications in biotechnology, medicine, and agriculture [73]. Modern tools like CRISPR-Cas systems offer high-precision gene editing guided by RNA, and transposons like Tn7 provide stable, site-specific integration of genes, making them invaluable for metabolic engineering and synthetic biology [74, 75]. Though recent research has broadened the genetic toolset for *Z. mobilis* and *C. sphaeroides*, facile tools and methods for delivering genetic loads to the genome and controlling gene expression still need expansion, especially for the *Novopshingobium sp.* A systematic stepwise approach is required for genome modification in a non-model microbe, like *Novosphingobium spp*. Based on experience working with *Z. mobilis* and *C. sphaeroides*, some of the factors mentioned here are necessary to expand to work with *Novosphingobium sp.* or other non-model bacteria.

### Genetically pliable strain for modification

One significant challenge in engineering bacterial strains, whether introducing new genes or removing genes, is the presence of restriction-modification (RM) systems that restrict heterologous DNA. Most bacteria have one or more RM systems that distinguish foreign DNA from native sequences, typically by differences in methylation patterns [76]. A widely used genome-modification approach involves adapting heterologous genes to the native methylation profile of the target species, often first in a restriction-deficient strain. Once adapted, these genes are transferred to the reference or wild-type strain for further study. In *E. coli*, restriction-deficient strains like DH5α or DH10β are used as cloning hosts, while MG1655 is the reference strain for genetic research [77]. Additionally, various non-methylating *E. coli* strains are employed to amplify vectors, helping them evade the host’s restriction-modification (RM) system when introduced into target strains.

In *Z. mobilis* ZM4, multiple RM systems have been identified [78]. The Rebase database [79] lists various RM systems across isolates of *Z. mobilis*, *C. sphaeroides*, and *N. aromaticivorans*, identified through bioinformatic and experimental studies. Among the RM systems in *Z. mobilis* ZM4, recent research has characterized a type I-F CRISPR system, two type I RM systems, and a type IV RM system similar to *mrr*. Restriction-deficient variants of these systems have shown improved compatibility with heterologous genes transferred from *E. coli* strains through conjugation [78]. Insight into these systems and their DNA recognition motifs provides valuable guidance for creating the next generation of *Z. mobilis* strains for efficient bioproduct synthesis.

Similarly, *C. sphaeroides* has multiple RM systems to defend against foreign DNA. Known endonucleases in *C. sphaeroides* strains, such as *RsaI* [80] (an isoschizomer of PvuI), *RshI* [81] (an isoschizomer of EcoRI), and *RsrI* [82, 83] have been documented in strains like *C. sphaeroides* 2.4.1 and *C. sphaeroides* RS630. PacBio long-read methylome sequencing has also revealed five restriction endonucleases in *C. sphaeroides* [84]. Additionally, *C. sphaeroides* mutants lacking the *rshI* gene show improved foreign gene uptake [85]. A comprehensive analysis of RM systems in non-model strains such as *N. aromaticivorans* will be crucial for advancing genetic engineering in these organisms.

###  Recombineering ability of strains

Genetic recombination plays a critical role in enhancing genetic diversity among bacterial strains, with recombination events impacting genomic stability, survival under DNA-damaging stressors, horizontal gene transfer, and evolutionary adaptation. While the recombination frequency and patterns can vary widely across strains due to complex genetic, environmental, and evolutionary interactions, *Z. mobilis* ZM4 has shown robust recombination capability in several studies [86]. The successful integration of an unstable vector into the ZM4 genome via single recombination events at a frequency of 0.3% [87] demonstrates its functional recombination machinery, making *Z. mobilis* ZM4 highly suitable for genetic engineering and biotechnological applications. Many studies have incorporated exogenous recombinases, such as *recECT* [88], lambda red recombinase [89], and FLP recombinase [90], indicating room to explore their impact on recombination efficiency further. Many other genes affecting native recombination systems in *Z. mobilis* ZM4 have also been studied. For instance, the role of the *himA* gene product in salt stress regulation, the role of *xerCD* and *recA* genes in DNA repair capabilities, and the recombination process. Several papers have explained homologous recombination-based genome modification, including horizontal gene transfer, in *C. sphaeroides* [91] and *N. aromaticivorans* [92], indicating robust recombination potential. The presence of the *recA* gene in both species further supports their potential for homologous recombination in DNA repair and genetic exchange [93]. The *recA* gene in *C. sphaeroides* encodes the *RecA* recombinase, a key protein involved in homologous recombination [94] and the SOS response following DNA damage such as UV exposure. *C. sphaeroides* possesses a complex genome with two chromosomes and multiple plasmids, featuring a high degree of gene duplication (~ 30%), which supports recombination processes. Experimental deletion of *recA* (Δ*recA* mutants) demonstrates that while RecA is not essential for aerobic growth, it is crucial for survival under DNA-damaging conditions. Δ*recA* mutants show markedly reduced viability following UV exposure, confirming RecA’s vital role in DNA repair. Genetic recombination in *Novosphingobium* underlies its metabolic adaptability, driven by horizontal gene transfer (HGT), genomic rearrangements, and advanced engineering tools. HGT enables the acquisition of key degradative genes like the *mlr* cluster, while plasmid integration and gene duplications enhance functional plasticity [95]. Regulatory elements like the *Rsh* regulon [96] and LuxR402 [97] influence genome dynamics and HGT potential via quorum sensing and cell envelope integrity. These features collectively support *Novosphingobium*’s recombination ability in metabolic engineering, particularly for degrading complex pollutants and optimizing lignin valorization in sustainable biotechnological applications [72, 98].

### Plasmid vectors used for genome engineering

####  Plasmid vectors for expression and transcriptional regulation studies

Genetic engineering frequently employs vectors to introduce specific genes or DNA sequences into target cells, allowing for gene manipulation in applications such as gene addition or deletion. Numerous plasmid vectors have been designed for bacterial genetics, incorporating essential elements like the origin of replication, mobilization elements, selectable markers (e.g., antibiotic resistance genes), and multiple cloning sites for foreign DNA insertion. A few are employed against the *Z. mobilis*,* C. sphaeroides*, and *Novosphingobium spp.* listed in Table 2. Among them, the broad-host-range plasmid *p*RL814, derived from *p*BBR, is commonly used in genetic engineering, especially with *Z. mobilis.* [99]. This plasmid includes features such as antibiotic resistance markers for selection, origins of replication for plasmid maintenance, a mobilization element for conjugation, a *lacI* and IPTG-inducible promoter, GFP, and multiple cloning sites. *p*RL814 has proven stable in *Z. mobilis* [87].

**Table 2:** Plasmid systems employed for various strategies in *Z. mobilis* and *Cereibacter*/*Rhodobacter sp*, with potential applicability to *Novosphingobium sp.*

S. No	Plasmid Name	Bacterial species	Strategies/ Mode of Transfer	Antibiotic Resistance	Description	Source
1	pZMO1 and other derivatives	*Z. mobilis*	Heterologous gene expression; Electroporation	Kan^R^; Cm^R^	Broad host range; replication origin of pBBR1; 1651 bp long with 38% G + C content & contains ORF of 1044 nucleotides. Stable in non-selective conditions	[100]
2	pZMOB04 and derivatives	*Z. mobilis* ATCC 10988	Heterologous gene expression; Electroporation	Kan^R^; Cm^R^	The broad host range, replication origin of pBBR1, 4023 bp size, can efficiently express *mCherry*, making it suitable for heterologous gene expression	[101]
3	pPK15534 and derivatives	*Z. mobilis*	Genome Engineering; Electroporation	Cm^R^	Broad host range; Unstable in *Z. mobilis* ZM4; Contains *gfp, mobilization* element; Low copy in *E. coli*; is used for insertional or deletion mutation	[102]
4	pRL814	*Z. mobilis*	Genetic manipulation; Conjugation	Sp^R^	Broad host range plasmid: IPTG–inducible; Stable in *Z. mobilis;* can be mobilized into *Z. mobilis* from *E. coli* WM6026 strains	[103]
5	pUC18 series vector	*Z. mobilis*	Transformation	Kan^R^	Not Stable in *Z. mobilis:* It has the resistance gene from pBBR1MCS-2	[104]
6	pZMO7	*Z. mobilis*	Electroporation	Cm^R^	Found in *Z. mobilis* with 4551 base pairs & it has significant use in heterologous protein expression	[105]
7	pHW20a	*Z. mobilis*	Gene manipulations; Transformation	Tet^R^	Higher efficiency in wild-type *Z. mobilis* ZM4 strains, stable, and ease of gene manipulation	[106]
8	pLAFR5 and derivatives	*Z. mobilis*	Gene expression; Conjugation [107]	Tet^R^	A low-copy-number cosmid vector was used to express heterologous genes in *Z. mobilis*	[108]
9	RSF1010-derived vectors	*Z. mobilis*	Gene expression; Electroporation [109]​​	Amp^R^	A plasmid with the tac promoter and *lacIq* repressor gene; Used to express *Z. mobilis* genes encoding *adhA*, *adhB*, and *pdc*, but it is unstable	[110]
10	pZB186	*Z. mobilis*	Transformation ​	Amp^R^	A mobilizable vector with MCS; *lacZ*α was used to construct pZB5 and pZB206 for *Z. mobilis* transformants, and to overexpress xylulokinase	[111]
11	pBR325	*Z. mobilis*	Transformation	Amp^R^; Tet^R^; Cm^R^	Broad host range; a natural plasmid of *Z. mobilis ATCC* 10988	[112]
12	pDS212	*Z. mobilis*	Transformation	Amp^R^; Tet^R^	A recombinant plasmid, ligated with EcoRI digests, was efficiently transformed into *E. coli* and transferred to *Z. mobilis* hosts, offering advantages for gene transfer system development	[112]
13	pLOI193, pLOI204	*Z. mobilis*	Gene expression; Transformation & Conjugation	Tet^R^	Stable plasmid for *Z. mobilis*	[113]
14	pRL27	*Z. mobilis;* *C. sphaeroides*	Conjugation [114]; Transduction ​​[115]	Kan^R^	A suicide vector designed for transposon delivery, ensuring a single transposon insertion per genome	[114]
15	pIND4	*Z. mobilis*; *C. sphaeroides*	Transformation ​[28, 116], Conjugation/ Electroporation [117]	Kan^R^	A *C. sphaeroides*-derived stable shuttle vector for *Escherichia coli, C. sphaeroides,* and *Z. mobilis*; IPTG-inducible	[118]
16	pBBR1MCS series	*Z. mobilis;* *C. sphaeroides*	Transformation; Gene expression	Tet^R^, Kan^R^, Amp^R^, and Cm^R^	small size (< 5.3 kb) and multiple cloning sites	Addgene (#85,168); [119]
17	pUC19	*Cereibacter sp.*	Gene expression vector	Amp^R^	Cloning vector	Addgene (#50,005); [104]
18	pRKPLHT and derivatives	*R. capsulatus; C. sphaeroides*	Transformation	Tet^R^; Kan^R^	Broad-host-range plasmid with an RK2 origin; ~ 11.4 kb size; used for gene expression in *R. capsulatus* and *C. sphaeroides*	[120, 121]
19	pEVS104	*Cereibacter* and *Novosphingobium sp*.	Conjugative helper vector for gene modification	Kan^R^	Conjugal helper plasmid that increases conjugation efficiency, does not replicate in recipient cells	Addgene (#207,393); [122]
20	pJMP8921 /8823/8930/8835 /8932	*Cereibacter* and *Novosphingobium sp*. [122]	Mobile-CRISPRi vector	Amp^R^	Mobilization vector; homologous recombination	Addgene (#220,903/#220,901/ #220,905/ # 220,902/ #220,906)
22	pK18*mobsacB*	*Novosphingobium sp*.	Integrative, mobilizable vector	Kan^R^	Mobilization vector; homologous recombination	[123]

The table outlines the different vectors used by the three microbes for genetic engineering. These vectors may be used or adapted for genetic engineering in *Novosphingobium sp*. Kan^R^: Kanamycin resistance gene; Cm^R^: Chloramphenicol resistance gene; Tet^R^: Tetracycline resistance gene; Amp^R^: ampicillin resistance gene; Sp^R^: spectinomycin resistance gene

Another frequently used plasmid, *p*IND4 (isopropyl-β-d-thiogalactopyranoside-inducible-expression plasmid), enables heterologous gene expression in *C. sphaeroides* and *Z. mobilis* through an IPTG-inducible promoter (P_A1/04/03_), ensuring controlled protein expression levels [124]. Initially developed for *C. sphaeroides*, *p*IND4 includes a kanamycin resistance cassette, a stable replicon of pMG160, *lacI*(q), and several expression elements for efficient use across alpha-proteobacteria [125]. The pMG170 is an *E. coli* shuttle cloning vector derived from *Rhodobacter blasticus*. This naturally occurring plasmid is stable and replicates effectively in other members of the *Rhodobacteraceae* family. *C. sphaeroides* maintains 18 to 23 copies of pMG160, while *E. coli* retains it at a high copy number [126]. The inducible promoter exhibits minimal leakiness in *C. sphaeroides* while enabling high protein production in *E. coli* [124]. It also contains a mobilization element facilitating the transfer from the *E. coli* donor strain S17-1 λpir [125] into *C. sphaeroides*. However, the conjugation of this plasmid into *Z. mobilis* and *N. aromaticivorans* has yet to be established.

Several vectors have been developed to sense byproduct synthesis activities, such as ethanol production, environmental cues, and promoter strength analysis [127]. Few studies have extensively worked on synthetic biology tools such as transcriptional biosensors, synthetic promoters, and high-throughput reporter assays to study gene regulation and protein function. While these work are not directly focused on microbial plasmid engineering, the techniques and principles developed from these work can be readily adapted for designing and optimizing plasmid-based gene expression systems in microbial hosts [128].

Fluorescent marker genes, such as g*fp* and *mCherry*, have been effectively used for promoter strength analysis in *Z. mobilis*, including vectors with a dual reporter system [127]. Additionally, a novel stilbene-responsive biosensor was created by mining the genome of *N. aromaticivorans* DSM 12444 [129]. This biosensor can differentiate resveratrol from its precursors and detect other stilbenes with resorcinol groups and cannabidiolic acid with a β-resorcylic acid functional group. It can also sense changes in resveratrol production within cells, potentially facilitating the metabolic engineering of microbial cell factories for stilbene and cannabinoid production [129].

#### Plasmid vectors for recombination-based genetic engineering

**Fig. 2:**
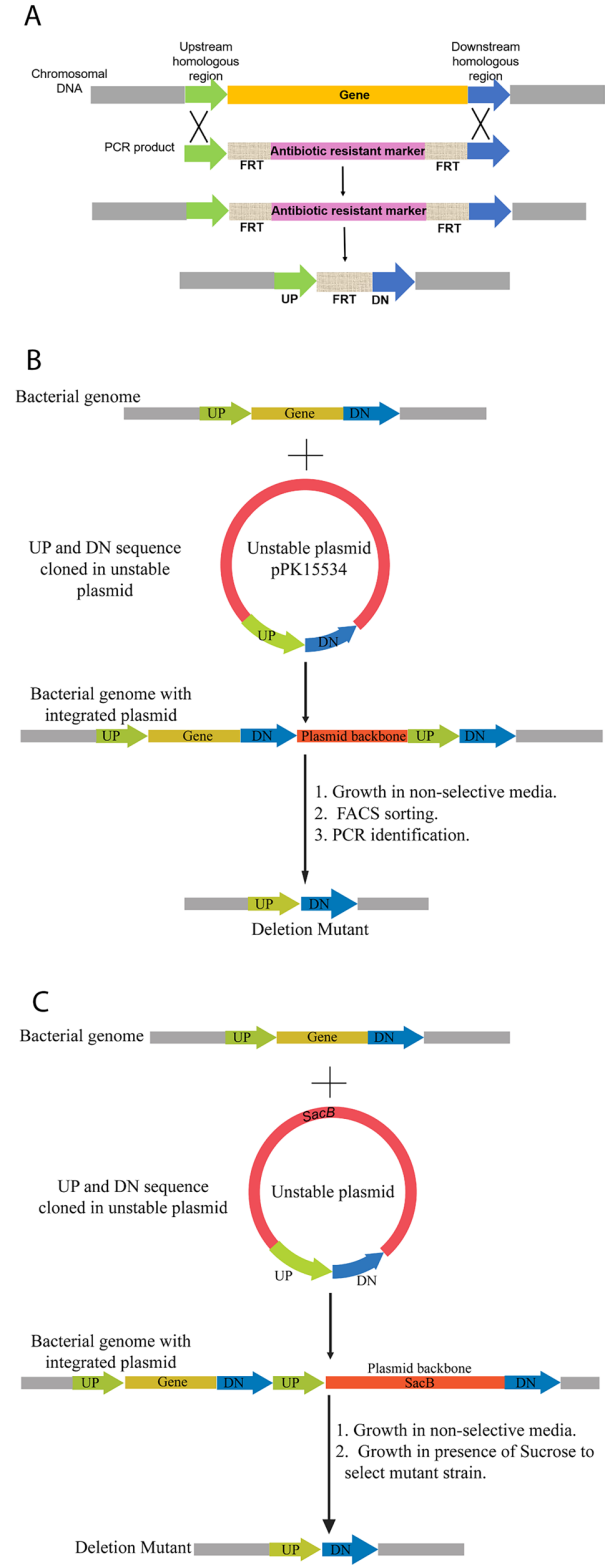
Strategies for Markerless Gene Deletion in Bacteria Using Homologous Recombination and Counterselection Systems. **A** Schematic representation of gene deletion using an FRT-flanked antibiotic resistance cassette and FLP recombinase system. An antibiotic resistance marker replaces the target gene through homologous recombination between upstream and downstream homologous regions. The resistance cassette is flanked by FRT (Flippase Recognition Target) sites. Following successful recombination and selection, expression of FLP recombinase mediates site-specific recombination between the FRT sites, excising the antibiotic marker and leaving behind a single FRT scar. This method facilitates the creation of markerless deletion suitable for multiple rounds of genome editing but leaves one FRT site in the genome. **B** A Schematic picture describes markerless gene deletion in *Z. mobilis*. Gene deletion in *Z. mobilis* is achieved by cloning 500 bp upstream (UP) and downstream (DN) of the target gene into the suicide plasmid pPK15534. The plasmid is introduced into the cells via conjugation, and a single crossover homologous recombination event is selected using an antibiotic resistance gene. Recombination can occur at the UP or DN regions; here, recombination at the DN region is shown. A second recombination event, detected by the loss of GFP fluorescence, results in plasmid excision. Deletion mutants are confirmed through PCR amplification. **C** Schematic representation of a two-step allelic exchange method using a *sacB*-based counterselection system for gene deletion in bacteria. The upstream (UP) and downstream (DN) flanking regions of the target gene are cloned into an unstable plasmid carrying the *sacB* gene. Upon transformation, homologous recombination integrates the plasmid into the bacterial genome via single crossover, creating a merodiploid. Subsequent recombination events under counter-selective conditions (e.g., sucrose selection against *sacB*) result in the excision of the plasmid backbone and *sacB*, generating a clean deletion mutant. This strategy facilitates scarless genome editing without leaving behind antibiotic resistance markers

Several approaches have been developed for gene inactivation, using either host-based recombination [130] or heterologous lambda recombinase-mediated recombination [89] to integrate an antibiotic-resistance gene flanked by host DNA in a suicide plasmid or linear DNA fragment, as demonstrated in *Z. mobilis* [131]. Plasmid curing has been a critical issue for such genome modifications, where an “unstable” or “suicide” vector is required to aid in curing plasmids after markerless genome modification (Fig. 2B).

Recently, a homologous recombination-based technique was introduced for markerless genome modifications in *Z. mobilis*. This two-step method initially integrates an engineered suicide plasmid (pPK15534) containing *Z. mobilis* targeting sequences into the genome through homologous recombination. The plasmid, equipped with GFP as a fluorescence marker, has two 500 bp homologous regions facilitating two recombination events. In the first event, the plasmid is integrated into the genome. In contrast, it is excised in the second, resulting in either a modified or wild-type genome at a specific ratio. The wild-type and deletion mutant genotypes are then differentiated by PCR amplification [102]. This system allows efficient gene addition or deletion without introducing antibiotic resistance genes into the *Z. mobilis* genome at the desired location, making it a versatile platform for genetic engineering applicable to other alphaproteobacteria. A key feature of this strategy is that GFP expressed from the suicide plasmid allows easy identification of cells that have lost the plasmid by using a fluorescence-activated cell sorter. This technique is efficient, adaptable, and applicable to other non-model bacteria [132]. A *sacB-*based counterselection marker has also been shown to be effective for plasmid curing (Fig. 2C). This approach has made the application feasible without the need for an expensive fluorescent-aided cell sorter, but *Z. mobilis* ZM4 is annotated to also have *sacB* [133], questioning the general utility of this approach for *Z. mobilis*.

### Transposon-based tool for genome modification

**Fig. 3:**
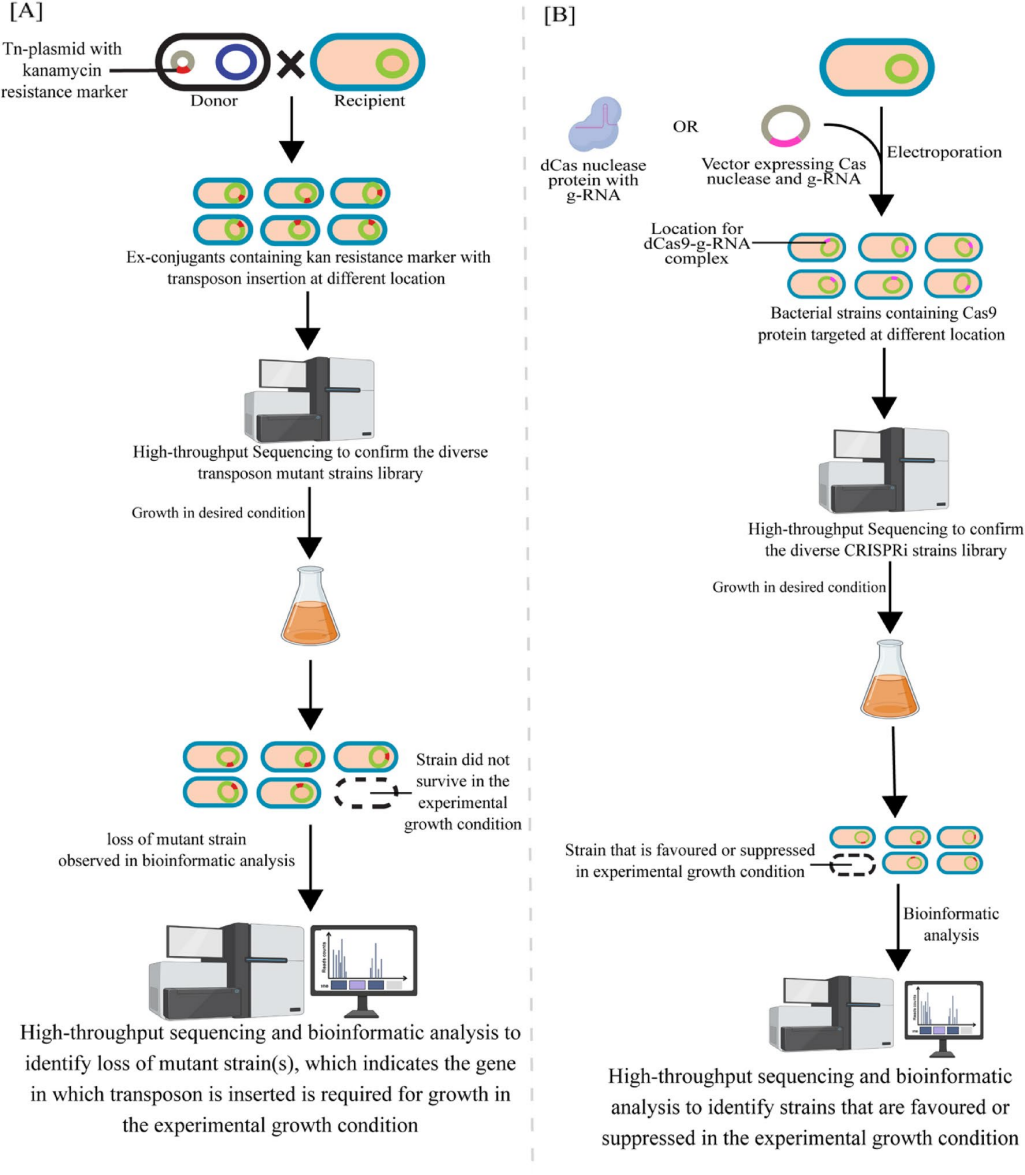
**A** Schematic representation of transposon mutagenesis in bacteria. The transposon-containing plasmid (Tn-plasmid) is introduced into the recipient bacterial strain using conjugation. This process requires a donor strain carrying the Tn-plasmid to be co-cultured with the recipient strain (target bacteria) for genetic material transfer. Following conjugation, ex-conjugants (recipient bacteria that have acquired the Tn-plasmid) are selected on kanamycin-containing agar plates, generating a pool of transposon mutants (mutant pool/library). This mutant pool is then cultured under optimal conditions, followed by genomic DNA extraction and sequencing to identify transposon insertion sites using bioinformatic analysis programs. The same mutant pool is subjected to specific growth conditions to assess gene essentiality. Loss of strain with transposon insertion in a gene indicates the essentiality of that gene for survival in the environment in which the library was grown. **B**. High-Throughput CRISPRi Screening Workflow to Identify Condition-Specific Bacterial Fitness Determinants. The figure illustrates a high-throughput CRISPR interference (CRISPRi) screening workflow to identify bacterial genes influencing growth under specific conditions. The process involves introducing dCas9 nuclease and guide RNAs (g-RNAs) into bacterial cells via electroporation, either as pre-formed complexes or through an expression vector, generating a diverse library of strains with different genomic targets. High-throughput sequencing confirms library diversity, and researchers then culture these strains under desired experimental conditions. Subsequent selection identifies favored or suppressed strains, and a final high-throughput sequencing coupled with bioinformatic analysis pinpoints the specific genes (targeted by the g-RNAs) that enhance or hinder bacterial growth under the tested conditions

Transposon mutagenesis has been extensively employed in bacteria to explore gene function, genetic pathways, and genome organization. This method can produce polar mutations, resulting in auxotrophic and antibiotic-sensitive mutants [134, 135]. The transposons Tn5, Tn7, and Tn10 are commonly used in bacterial genetics. While Tn5 and Tn10 insert randomly throughout the genome, Tn7 integrates at specific attachment sites. Recent advancements in high-throughput nucleic acid sequencing have significantly improved our ability to analyze the outcomes of random mutagenesis induced by these transposons. Tn5 transposon mutagenesis combined with high-throughput sequencing (Tn-Seq or TraDIS) provides a powerful tool in genetics research for identifying essential genes and understanding their functions within an organism’s genome [114]. Unlike classical genetics, which often relies on targeted gene deletions, Tn-Seq leverages random transposon insertions, usually in single copies per genome. Strains with insertions in essential genes are expected to lose viability in conditions requiring those genes for growth or fitness [114]. By evaluating transposon insertion frequency across a genome under a specific growth condition, Tn-Seq facilitates functional genome analysis. This method has been applied to identify essential genes and assess their roles in growth and fitness in *C. sphaeroides* [136] and *Z. mobilis* [114]. A related approach, CRISPR interference (CRISPRi), has also been developed for similar functional studies (Fig. 3).

### CRISPR tools used for genome engineering

#### CRISPR-based tools for targeted double-strand nicks and genome engineering

**Fig. 4:**
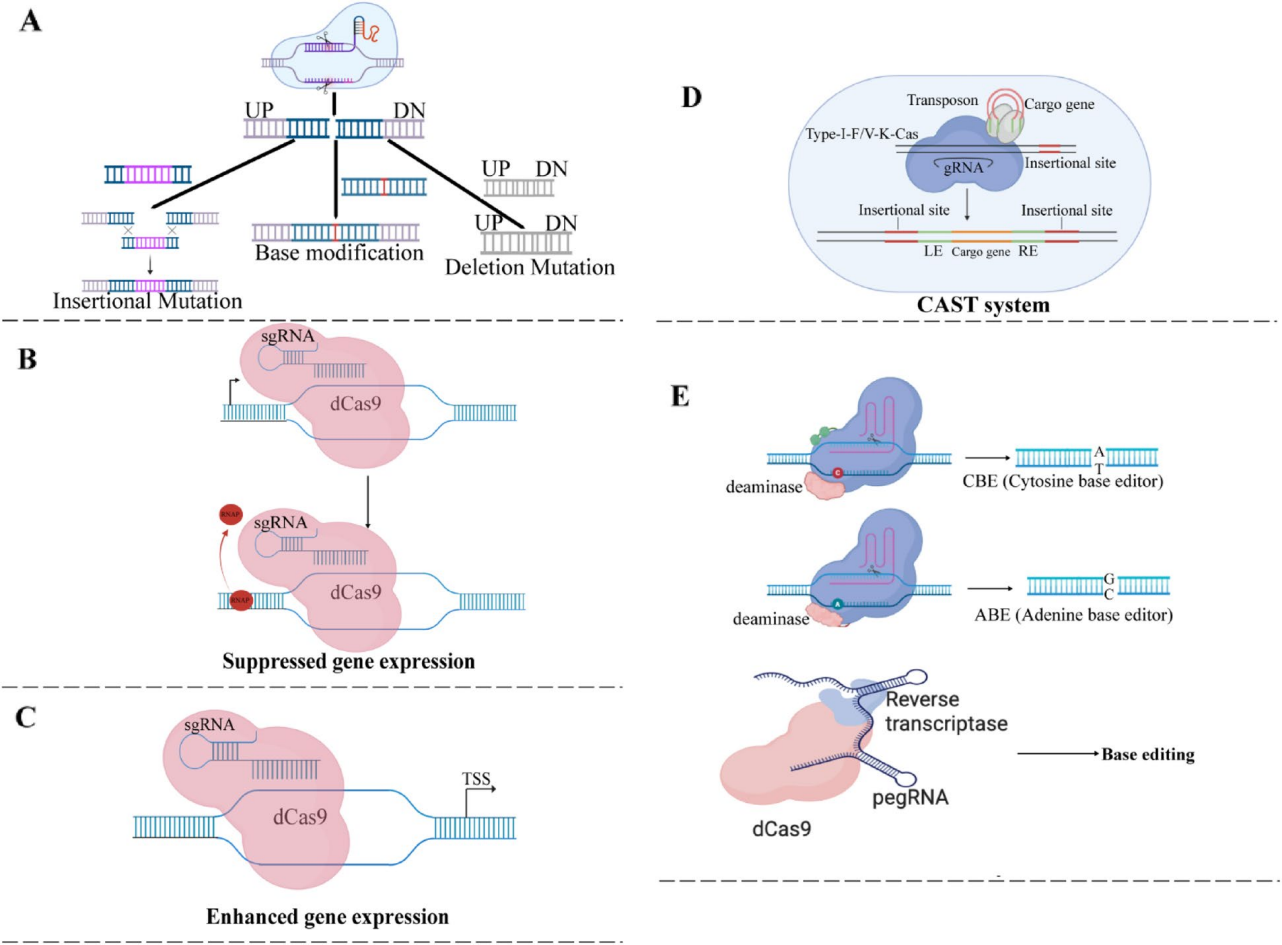
CRISPR–Cas-Based Strategies for Microbial Genome Editing, Gene Deletion, Replacement, and Transcriptional Suppression (**A**) Illustration of Cas9-mediated genome editing. Wild-type Cas9 binds to a single-guide RNA (sgRNA) that directs it to a specific DNA sequence adjacent to a PAM site. Cas9 introduces a double-strand break at this target site. The break is then repaired through homology-directed repair (HDR), incorporating an exogenous DNA template carrying the desired modification, such as a marker gene insertion, base change, or deletion, into the chromosome, resulting in targeted insertion, modification, or deletion mutations. (**B**) Illustration of dCas9-mediated gene regulation. dCas9 (dead Cas9) lacks nuclease activity but retains the ability to bind target DNA through sgRNA. By occupying the DNA, it blocks RNA polymerase (RNAP) access, thereby preventing transcription and causing gene repression. This figure contrasts Cas9-based genome editing (DNA cutting and modification) with dCas9-based transcriptional repression (blocking gene expression without DNA cleavage). (**C**) Illustration of CRISPR activation (CRISPRa) using a dCas9-sgRNA complex. A single-guide RNA (sgRNA) directs catalytically inactive Cas9 (dCas9), fused to a transcriptional activation domain, to a target DNA region near the transcriptional start site (TSS) of a gene. The binding of this complex recruits transcriptional machinery, enhancing gene expression without altering the DNA sequence. (**D**) Schematic overview of the CRISPR-associated transposase (CAST) system. The Cascade complex, composed of Cas6, Cas7, and Cas8, is guided by CRISPR RNA (crRNA) to a specific DNA target site. Upon recognition, the accessory protein TniQ connects Cascade to the transposition machinery, which includes transposase components. These mediate the integration of a donor DNA element into the host genome, flanked by transposon ends (left end, LE; right end, RE). The donor cassette contains a cargo gene, such as a selectable marker or gene of interest for functional expression. This RNA-guided, site-specific integration occurs without double-strand breaks, providing a versatile genome engineering tool suitable for bacterial hosts like *Z. mobilis*,* C. sphaeroides*, and *N. aromaticivorans*. (**E**) Mechanism of Programmable Base Editing and Prime Editing for Precision Genome Engineering. This diagram depicts programmable base editing, allowing precise nucleotide changes without introducing double-strand breaks. In cytosine base editors (CBEs), a catalytically impaired Cas9 (dCas9 or Cas9 nickase) is fused to a cytosine deaminase that converts cytosine (**C**) to uracil (U), leading to a C-G to T-A transition following DNA replication or repair. Adenine base editors (ABEs) use a TadA-TadA deaminase fusion to convert adenine (**A**) into a base read as guanine **G**, resulting in an A-T to G-C substitution. Cytosine base editors often include an uracil glycosylase inhibitor (UGI) to block base excision repair. The sgRNA binding site and the deaminase’s effective range determine the editing window. This technology enables efficient, scarless point mutations, providing a powerful alternative to traditional gene knockouts, especially in organisms with limited double-strand break repair mechanisms. The bottom panel depicts promoter engineering using dCas9 fused with a reverse transcriptase and a prime editing guide RNA (pegRNA). This enables precise base or prime editing of promoter regions to modulate gene expression

High-throughput CRISPR-based genome editing techniques have become powerful tools for precise gene engineering in various microbes, facilitating iterative addition and removal of exogenous DNA containing marker genes (Fig. 4A). These techniques are widely recognized in molecular biology for their precision and efficiency [122]. Despite widespread success in model organisms, the application of these technologies in non-model microbes remains limited. While multiple CRISPR methods have been developed for *Z. mobilis* [137], fewer advances have been made for *C. sphaeroides* and *N. aromaticivorans*. Gene deletions typically employ self-targeting or suicide plasmids encoding mini-CRISPR arrays and Cas protein that induce site-specific DNA cleavage followed by repair through microhomology-mediated end joining (MMEJ). This approach has successfully enabled gene and ample fragment knockouts, improving efficiency through optimized guide RNA design and target site selection [138]. Plasmid curing, essential for iterative editing and strain development, is generally achieved by subculturing in antibiotic-free media or using temperature-sensitive [139] and suicide plasmids [140].

CRISPR systems are broadly classified into two major classes based on complexity and signature Cas proteins: Class 1, which uses multi-subunit complexes (types I, III, IV), and Class 2, which employs single, crRNA-guided effector proteins (types II, V, VI) [141, 142]. These systems introduce site-specific DNA double-strand breaks (DSBs) at target loci guided by engineered RNAs, triggering endogenous DNA repair mechanisms such as non-homologous end joining (NHEJ) or homologous recombination (HR) [143, 144].

In many prokaryotes, including *Z. mobilis*, NHEJ is inefficient or incomplete, causing reliance on HR and donor DNA templates for precise genome editing [1]. Genome editing in *Z. mobilis* has been effectively accomplished using endogenous type I-F CRISPR-Cas systems and an exogenous type II CRISPR-Cas9 system [145, 146]. These advancements have enabled multiplex genome editing, allowing simultaneous modifications at multiple loci without iterative, marker-dependent steps [147]. For example, applying the *Streptococcus pyogenes* CRISPR/Cas9 system in *Z. mobilis* ZM4 for targeted genome editing and native plasmid elimination [148]. However, Cas12a (Cpf1), a Type V RNA-guided endonuclease, has gained prominence for microbial genome editing due to its reduced cytotoxicity compared to Cas9, recognition of T-rich PAMs, and staggered DNA cleavage pattern [149, 150]. Conversely, Cas9 targets G-rich PAMs and generates blunt-ended DSBs [151]. Multiplex genome editing, enabled by Cas12a’s ability to process its own crRNA array independently of tracrRNA, allows simultaneous modification of multiple loci, significantly accelerating microbial engineering [147, 152]. For example, CRISPR-Cas12a recombineering in *Z. mobilis* achieves one-step gene deletions or insertions with efficiencies near 90%, enabling metabolic redirection such as diverting carbon flux from ethanol to lactate [153].

#### CRISPR-based tools for transcriptional regulation

CRISPR-based transcriptional regulation tools such as CRISPR interference (CRISPRi) and CRISPR activation (CRISPRa) have emerged as powerful platforms for precise and reversible gene expression control without inducing double-stranded DNA breaks. These systems utilize a catalytically inactive Cas9 (dCas9) protein guided by single-guide RNAs (sgRNAs) to target specific genomic loci [154] (Fig. 4C). In CRISPRi, dCas9 physically obstructs transcription initiation or elongation, often enhanced by fusion to repressor domains like KRAB [155]. Conversely, CRISPRa fuses dCas9 to transcriptional activators (e.g., VP64, p300, or SAM system), promoting transcription from targeted promoter or enhancer regions [156]. These programmable tools allow modulation of gene expression in a tunable and non-destructive manner, making them especially valuable for studying essential genes and complex regulatory networks.

Recent advances have enabled deploying CRISPRi systems in non-model organisms such as *Z. mobilis*, *C. sphaeroides*, and *N. aromaticivorans*. In *Z. mobilis*, an optimized CRISPRi system employing dCas9 was developed to achieve inducible, titratable repression of essential and nonessential genes. This system has proven effective for several bacterial species including *Z. mobilis* and *E. coli*, underscoring its cross-species potential [157] (Fig. 4B). The ability to modulate gene expression with high precision in *Z. mobilis* expands the genetic toolbox for metabolic engineering and functional genomics in industrial microbes.

Parallel efforts in *C. sphaeroides* and *N. aromaticivorans* have demonstrated the utility of synthetic biology tools to integrate CRISPRi systems stably and functionally [158]. Tn7-mediated chromosomal integration enabled stable insertion of engineered DNA constructs into these Alphaproteobacteria. At the same time, a synthetic promoter library screening identified IPTG-inducible promoters capable of achieving up to ~ 15-fold induction in *N. aromaticivorans* and ~ 5-fold in *C. sphaeroides*. Combined with CRISPRi constructs, these tools allowed for ~ 10-fold repression of target genes, including essential loci, facilitating pathway modulation and gene function studies in species previously considered genetically intractable [158].

Together, these developments illustrate a growing capability to adapt CRISPR-based transcriptional control systems to diverse bacterial chassis, paving the way for sophisticated genetic manipulations in biotechnologically relevant Alphaproteobacteria.

####  CRISPR-based tools for targeted single-strand nicks and genome engineering

CRISPR-derived base editors, including cytosine base editors (CBEs) and adenine base editors (ABEs), represent a next-generation genome engineering strategy that enables precise single-nucleotide substitutions without introducing double-strand breaks (DSBs). These systems fuse a catalytically impaired Cas protein (e.g., dCas9 or Cas9 nickase) with a deaminase enzyme to induce site-specific C-G to T-A or A-T to G-C conversions, respectively [159].

In *C. sphaeroides*, both CBEs and ABEs have been successfully deployed to achieve efficient C-to-T and A-to-G editing, demonstrating the feasibility of precise and multiplexable single-base modifications in this non-model Alphaproteobacterium [160] (Fig. 4E- Left panel). This work marks a significant step toward high-resolution genetic manipulation in *C. sphaeroides*. These tools offer enhanced precision and versatility for introducing targeted edits without relying on homologous recombination or donor templates. However, these tools are underutilized in *Z. mobilis*, *C. sphaeroides*, and *N. aromaticivorans*.

####  RNA-guided DNA insertion with CRISPR-associated transposase (CAST)

CASTs (CRISPR-associated transposases) are specialized CRISPR systems that combine CRISPR-Cas proteins with transposases to enable RNA-guided, site-specific DNA insertions without requiring double-strand breaks (DSBs) or homology-directed repair (HDR) [161]. CAST systems are naturally occurring CRISPR-transposon hybrids found in some bacteria [162]. They allow programmable DNA integration using a guide RNA, similar to traditional CRISPR systems, but instead of cutting DNA, they insert genetic material at specific sites with high precision. Figure 4D illustrates that the CAST systems typically include a CRISPR-Cas protein, usually a variant of Cas12k (Type V-K) or Cas8/Cas5/Cas6 complex (in Type I systems), a transposase complex, such as TnsA, TnsB, and TnsC or TniQ, an RNA (gRNA) to direct the system to the target DNA sequence, and a DNA (cargo gene), often flanked by transposon ends, to be inserted into the genome (Fig. 4D). The gRNA directs the Cas protein to a target site. The transposase components recognize this site and catalyze the integration of the donor DNA into the genome at or near the target. Unlike traditional CRISPR methods, no DSBs are made, and no homologous arms are needed. CASTs are still in the early stages of synthetic biology and genome engineering, and the development of their potential is being actively explored [163]. They have been demonstrated in *E. coli*, and researchers are working to expand their use to other bacteria and eukaryotes.

#### Pan-genome functional gene analysis using CRISPRi and transposon-based tools

Screening gene perturbation libraries against stressors or experimental conditions can reveal valuable engineering targets; however, follow-up validation to rule out false positives is often time-consuming and resource-intensive. Recently, an orthogonal gene perturbation strategy (CRISPRi–TnSeq) combining Tn-Seq (Fig. 3A) and CRISPRi (Fig. 3B) has been developed in *Z. mobilis* [164]. These two methods complement each other; CRISPRi allows targeted and reversible repression of genes, including essential ones, while Tn-Seq offers broad, unbiased mutagenesis for comprehensive genome-wide functional analysis. The CRISPRi–TnSeq method provides high sensitivity and scalability, with minimal polar effects, and is especially effective for dissecting gene networks and prioritizing synthetic lethal interactions. CRISPRi–TnSeq is thus a powerful tool to systematically explore gene function and interaction landscapes in microbial genomes [165, 166].

#### PEG RNA-based Cas9 systems (Prime editing guide RNA or Peg RNA)

PEG RNA-based Cas9 systems, more commonly called prime editing, use a specialized guide RNA known as prime editing guide RNA (pegRNA) [167]. This system combines a Cas9 nickase (which makes a single-strand cut instead of a double-strand break) fused to a reverse transcriptase enzyme. The pegRNA both directs the Cas9 to the target site and provides a template for the desired DNA edit, enabling precise insertions, deletions, or base substitutions without causing double-strand breaks or relying on donor DNA templates [168, 169] (Fig. 4E- right Panel). This system is a promising new CRISPR technology for precise genome editing, but its application in *Z. mobilis*, *C. sphaeroides*, and *N. aromaticivorans* has not been reported yet.

### ZFNs and TALENs for targeted double-strand break and genome modification

Similar to CRISPR-Cas nucleases, zinc finger nucleases (ZFNs) and transcription activator-like effector nucleases (TALENs) are programmable nuclease-based tools that induce targeted double-strand breaks (DSBs), enabling precise genome modifications via cellular repair pathways such as HR or NHEJ, leading to insertions or deletions that will allow precise genome modifications with high efficiency [170]. In both systems, DNA recognition is mediated by engineered proteins, zinc finger proteins (ZFPs) in ZFNs and transcription activator-like effectors (TALEs) in TALENs. At the same time, the fused FokI endonuclease domain catalyzes DNA cleavage [171]. Despite their advantages as gene editing tools, ZFNs and TALENs have not yet been extensively developed or applied for genome engineering in *Z. mobilis*, *C. sphaeroides*, or *Novosphingobium spp*., due to complex protein design, higher costs, off-target risks, and limited capacity for multiplex editing.

## Conclusion

This review highlights the bioenergy potential of *Zymomonas*, *Cereibacter*, and *Novosphingobium* species by emphasizing their unique metabolic traits, advances in genome-scale modeling, and development of genetic tools. Although many tools exist for other microbes, they often need to be adapted for use in these species. Their close phylogenetic relationships suggest that engineering strategies and tools developed for one organism can be transferred and modified with minimal effort for others, speeding up strain development. Broadening the application of these emerging technologies will be critical to fully harnessing the metabolic potential of these organisms. Furthermore, the distinct capabilities of each microbe, when combined synergistically, could offer a powerful platform for producing a diverse range of bioenergy compounds.

## Supplementary Information

Below is the link to the electronic supplementary material.

Supplementary Material 1

## Data Availability

All datasets generated or analyzed during this study are included in this published article [and its supplementary information files].

## Abbreviations

CI: Chromosome I
CII: Chromosome II
FLP: recombinase- Flippase recombinase
GFP: Green fluorescent protein
RBS: Ribosomal binding site
Sp.: Species
TraDIS: Transposon directed insertion-site sequencing
