# Supplementary material for: Genetic tools for engineering Zymomonas mobilis, Cereibacter sphaeroides and Novosphingobium aromaticivorans to improve production of bioenergy compounds
Source: Microb Cell Fact. 2025 Nov 14;24:239. doi: 10.1186/s12934-025-02845-3 (PMC12619356; doi:10.1186/s12934-025-02845-3)

**Figure Legend**

**Fig. S1. ED and Heterologous Pathways in *Z. mobilis* for Glucose Utilization and Biofuel Production.** The inherent ED (Entner-Doudoroff) pathway and heterologous pathway (shown as dashed line) in *Zymomonas mobilis* that facilitate the utilization of glucose and production of ethanol and isobutanol. GK: Glucose kinase; GPDH: Glycerol-3-phosphate dehydrogenase; PGL: 6-phosphogluconolactonase; EDD: 6-phosphogluconate dehydratase; EDA: 2-keto-3-deoxygluconate-6-phosphate aldolase; GAPDH: Glyceraldehyde-3-phosphate dehydrogenase; PGK: Phosphoglycerate kinase; PGM: Phosphoglycerate mutase; ENO: Enolase; PYK: Pyruvate kinase; PDC: Pyruvate dehydrogenase complex; ADH: Alcohol dehydrogenase; AIsS: Acetolactate synthase; KARI: Ketol-acid reductoisomerase; DHAD: Dihydroxy acid dehydratase; KIVD: α-ketoisovalerate decarboxylase; KDPG: 2-keto-3-deoxy-6-phosphogluconate; ATP: Adenosine triphosphate; ADP: Adenosine diphosphate; NADP: Nicotinamide adenine dinucleotide phosphate; NADH: Nicotinamide adenine dinucleotide

**Fig. S2. MEP and Heterologous MVA Pathways in Rhodobacter sphaeroides for Isoprenoid Biosynthesis.** Illustration of MEP (Methylerythritol phosphate pathway) and Heterologous MVA (Mevalonate) pathways (with dotted arrow) utilized by *Rhodobacter sphaeroides* for isoprenoid synthesis. G-3-P: Glyceraldehyde-3-phosphate; PYR: Pyruvate; DXP: 1-deoxy-D-xylulose-5-phosphate; MEP: 2-C-methyl-D-erythritol-4-phosphate; CDP-ME: 4-diphosphocytidyl-2-C-methyl-D-erythritol; CDP-MEP: 4-diphosphocytidyl-2-C-methyl-D-erythritol 2-phosphate; MEcPP: 2-C-methyl-D-erythritol 2,4-cyclodiphosphate; HMBPP: 4-hydroxy-3-methyl-but-2-enyl diphosphate; Ac-CoA: Acetyl-Coenzyme A; AA-CoA: Acetoacetyl-CoA ; HMG-CoA: 3-hydroxy-3-methylglutaryl coenzyme A; MVA: Mevalonate; MVA-P: Mevalonate-3-phosphate; MVA-PP: Mevalonate-3,5-bisphosphate; IPP: Isopentenyl diphosphate; DMAPP: Dimethylallyl diphosphate; GPP: Geranyl pyrophosphate; FPP: Farnedyl pyrophosphate; GGPP: Geranylgeranyl pyrophosphate

**Figures**

Supplementary Fig.1


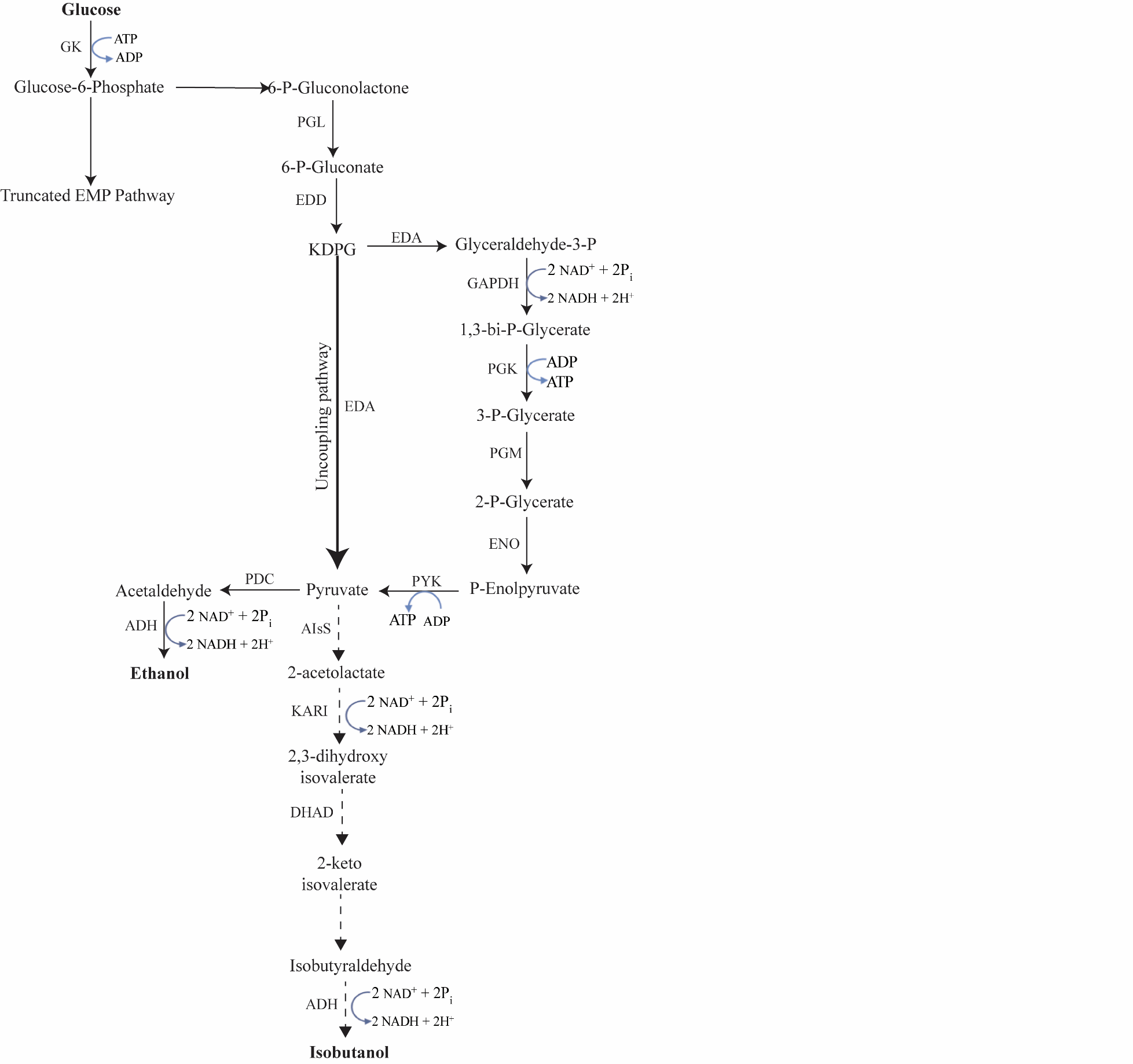


Supplementary Fig.2


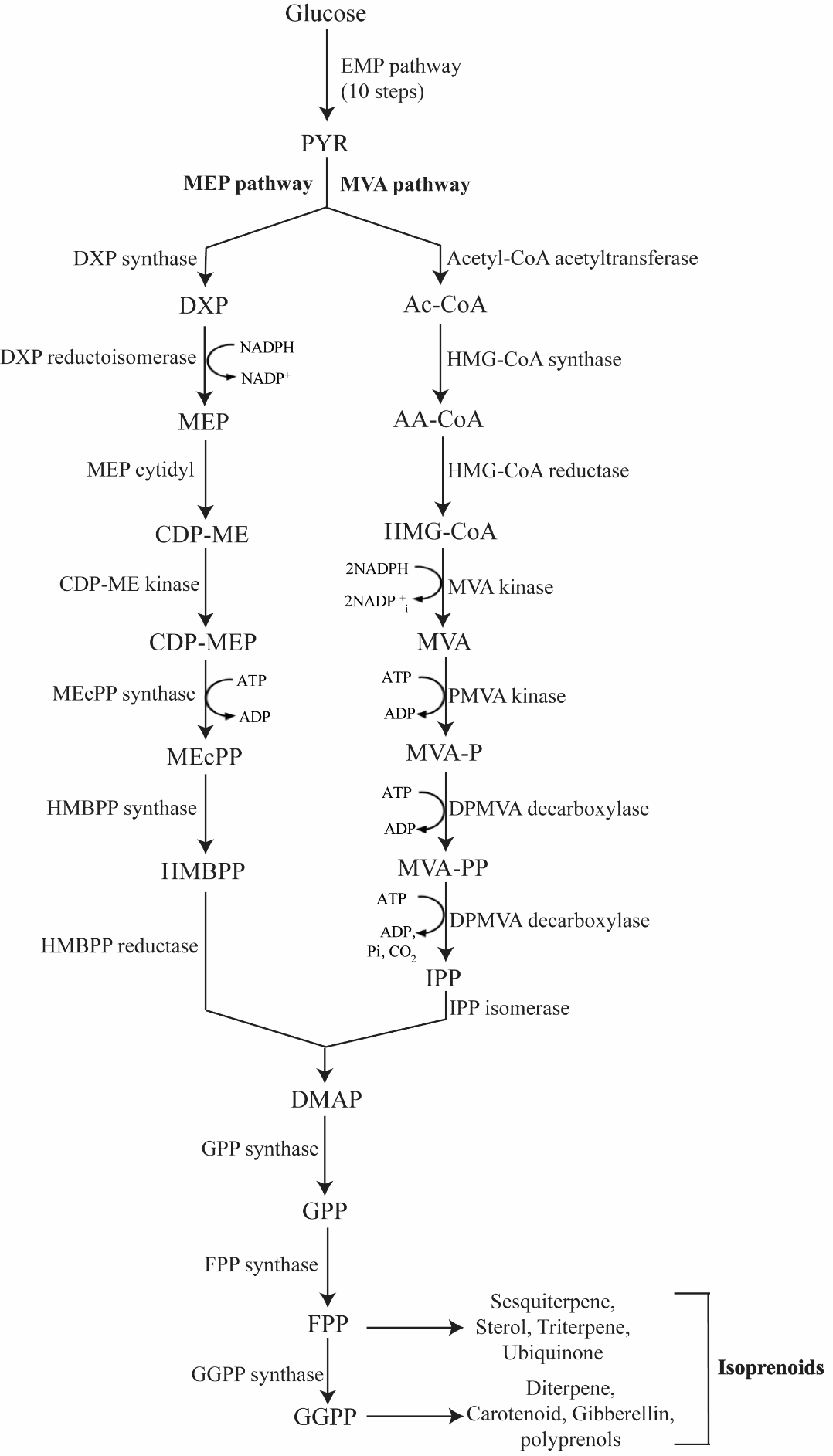

Supplement: Supplementary file 1 — Supplementary Material 1 [file 12934_2025_2845_MOESM1_ESM.docx]
